# Residual inflammatory risk and clinical outcomes after contemporary percutaneous coronary intervention: a systematic review and meta-analysis

**DOI:** 10.1038/s41598-026-39691-1

**Published:** 2026-02-12

**Authors:** Francisco José Romeo, Michele Golino, Matteo Morello, Francesca Maria Di Muro, Francesco Moroni, Marco Giuseppe del Buono, Giuseppe Biondi-Zoccai, Antonio Abbate

**Affiliations:** 1 Department of Cardiology, University of Miami Miller School of Medicine, Jackson Memorial Hospital, 1120 NW 14th St, Miami, FL 33136 USA; 2 Pauley Heart Center, Virginia Commonwealth University, Richmond, VA USA; 3 Department of Cardiology and Robert M. Berne Cardiovascular Research Center, The University of Virginia, Charlottesville, VA USA; 4 Department of Molecular and Translational Medicine, University of Brescia, Brescia, Italy; 5 The Zena and Michael A. Wiener Cardiovascular Institute, Icahn School of Medicine at Mount Sinai, New York, NY USA; 6 Department of Experimental and Clinical Medicine, School of Human Health Sciences, Careggi University Hospital, University of Florence, Florence, Italy; 7 Department of Cardiology, Agostino Gemelli University Polyclinic, Rome, Italy; 8 Department of Medical-Surgical Sciences and Biotechnologies, Sapienza University, Latina, Italy; 9 Maria Cecilia Hospital, GVM Care & Research, Cotignola, Italy

**Keywords:** Residual inflammatory risk, C-reactive protein, Percutaneous coronary intervention, Meta-analysis, Cardiology, Interventional cardiology, Chronic inflammation

## Abstract

Elevated low-density lipoprotein cholesterol (LDL-C) and subclinical inflammation - measured with high-sensitivity C-reactive protein (hsCRP) - contribute to atherosclerosis progression. Despite medical therapy, high-risk patients may still have residual cholesterol risk, residual inflammatory risk (RIR), both, or neither. We aimed to study the impact of RIR in patients undergoing contemporary percutaneous coronary intervention (PCI). A comprehensive search of Pubmed, EMBASE, Cochrane and MEDLINE was performed up to December 2024. Only studies including patients with coronary artery disease undergoing PCI were considered. Inflammatory burden was evaluated with two hs-CRP measurements at baseline and follow-up (> 4 weeks apart). High RIR was defined as hsCRP ≥ 2 mg/L at 1-month follow-up. The risk ratio (RR) with a 95% confidence interval (CI) was computed using a random-effect model. We identified five studies enrolling 13,604 patients, including 5,833 with high RIR at 30 days post–PCI. At 12-month follow-up, persistent high RIR was associated with an increased risk of major adverse cardiovascular events (MACE) (random-effects RR = 1.64, 95% CI 1.33–2.03; I² = 80.2%). High RIR was also associated with increased all-cause mortality (random-effects RR = 3.25, 95% CI 2.49–4.25; I² = 67%), non-fatal myocardial infarction (random-effects RR = 1.46, 95% CI 1.00–2.12; I² = 73%), and non-fatal stroke (random-effects RR = 1.64, 95% CI 1.14–2.37; I² = 0%). Sensitivity analyses, including Baujat plots and leave-one-out analyses, identified a single study as a major contributor to heterogeneity; exclusion of this study substantially reduced heterogeneity without materially altering the direction or magnitude of the associations. In patients undergoing contemporary PCI, a high RIR was associated with a significantly higher risk of adverse cardiovascular outcomes. This data could help tailor lipid-lowering and anti-inflammatory therapies in this high-risk population.

**Supplementary Information:**

The online version contains supplementary material available at 10.1038/s41598-026-39691-1.

## Introduction

Atherosclerosis, a chronic inflammatory disease of the arterial wall, is a principal driver of coronary artery disease (CAD) and its clinical manifestations, including myocardial infarction and ischemia. The pathogenesis of atherosclerosis is fundamentally linked to endothelial dysfunction, lipid accumulation, and a sustained inflammatory response mediated by both innate and adaptive immune systems^1^. Over time, this process results in the formation of atherosclerotic plaques, some of which may become unstable and rupture, precipitating acute coronary syndromes (ACS). In patients naïve of medical therapy, it is known that both inflammation and hyperlipidaemia contribute equally to the risk of atherothrombotic events^2^. In addition, in patients on statin therapy, circulating biomarkers including high-sensitivity reactive protein (hsCRP) as well as interleukin-6 (IL-6) are associated with “excess” risk of cardiovascular events in patients with otherwise “normal” cholesterol levels and other traditional risk factors. After initiating aggressive statin therapy, high-risk atherosclerosis patients can be classified as having residual cholesterol risk (on-treatment low-density lipoprotein cholesterol [LDL-C] ≥ 70 mg/dL), residual inflammatory risk (on-treatment high-sensitivity C-reactive protein[hsCRP] ≥ 2 mg/L), both conditions, or neither. Residual inflammatory risk (RIR) has been proposed as a stronger predictor of cardiovascular (CV) events in comparison with residual cholesterol risk^3,4^. In addition, a recent collaborative analysis of 31,245 patients with-or at high risk of atherosclerotic disease, on contemporary statin therapy, demonstrated that hsCRP was a stronger predictor for risk of future cardiovascular events and death than cholesterol assessed by LDL-C^5^. Patient undergoing percutaneous coronary intervention (PCI) undergo assessment of residual cholesterol risk according to current guidelines^6,7^. However, the prevalence of high RIR in PCI-treated patients is not well established and assessment of RIR is not recommended in current guidelines. In this systematic review and meta-analysis of patients undergoing PCI, we aim to determine the prevalence of high RIR 1-month after PCI as well to evaluate the association with adverse clinical outcomes at 1-year follow-up.

## Materials and methods

### Research selection and data extraction

This meta-analysis was performed conformed with standards set forth by the Preferred Reporting Items for Systematic Reviews and Meta-analyses (PRISMA) statement^8^ and registered in PROSPERO [CRD42023413348]. In addition, this systematic review and meta-analysis was conducted following the PRISMA 2020 guidelines (Preferred Reporting Items for Systematic Reviews and Meta-Analyses). The checklist was used to ensure transparency and completeness in reporting. We adhered to the PRISMA 2020 guidelines in the development of the research question, eligibility criteria, information sources, study selection, data extraction, synthesis methods, and assessment of risk of bias. A copy of the PRISMA 2020 checklist is available as Supplementary Tables 1-PRISMA 2020 checklist.

A comprehensive search of the literature including Pubmed, EMBASE, Cochrane and Scopus were performed through February 2025. The search strategy combined Medical Subject Headings (MeSH) terms and free-text keywords, including: “Percutaneous Coronary Intervention” OR PCI) AND (“residual inflammatory risk” OR inflammation OR “inflammatory biomarkers”) AND (“C-Reactive Protein” OR CRP OR “Interleukin-6” OR IL-6 OR TNF-α) AND (“cardiovascular disease” OR “coronary artery disease”. Boolean operators (AND, OR) were applied to optimize the search sensitivity, and the strategy was adapted for each database. Reference lists of included studies and related reviews were manually screened to identify additional eligible studies. No language restrictions were applied. Residual inflammatory risk was defined according to study-specific hsCRP thresholds, reflecting population-specific distributions and prior validation within each cohort. No attempt was made to harmonize hsCRP cutoffs across studies. Two independent reviewers (FR and MG) performed title and abstract screening, followed by full-text review to confirm eligibility. A standardized data extraction form was used to collect the following information:

- Study characteristics (authors, publication year, study design, and location).
- Patient demographics and baseline clinical characteristics.
- Definitions and thresholds for RIR (e.g., hsCRP cutoff values).
- PCI procedural details and adjunctive therapies.
- Outcomes of interest, including MACE and its individual components, as well as follow-up duration.

Discrepancies between reviewers were resolved by discussion or consultation with a third reviewer (MM).

### Inclusion criteria

Studies were eligible for inclusion if they met the following criteria:

1. Reported on Residual Inflammatory Risk (RIR), defined by inflammatory biomarkers such as high-sensitivity C-reactive protein (hsCRP) or equivalent measures at baseline and 1 month (± 5 days) after PCI.
2. Included adult patients (> 18 years) undergoing PCI for stable coronary artery disease (CAD) or acute coronary syndromes (ACS).
3. Reported clinical outcomes, including major adverse cardiovascular events (MACE), all-cause mortality, non-fatal myocardial infarction, non-fatal stroke, or major bleeding. The primary outcome was MACE, defined as a composite of all-cause mortality, non-fatal myocardial infarction (MI), and non-fatal stroke. Major bleeding events were analyzed separately and were not included in the MACE definition. When available, non-fatal MI corresponded to spontaneous (type 1) events as reported by individual studies.
4. Were randomized controlled trials (RCTs), prospective or retrospective cohort studies, or case-control studies.

### Exclusion criteria

Studies were excluded if they:

1. Did not provide quantitative data on RIR or relevant clinical outcomes.
2. Were reviews, editorials, conference abstracts, or case reports.
3. Had overlapping or duplicate patient populations.
4. Did not provide two data points of hsCRP and LDL-C at baseline and 1 month follow-up.

### Quality assessment of included studies

Risk of bias for non-randomized studies was evaluated using the ROBINS-I tool across seven domains^9^. For this analysis, “intervention” referred to exposure to high versus low hsCRP levels following PCI. Two reviewers (FR and MG) independently assessed each domain, with discrepancies resolved by a third reviewer (MM). The overall risk of bias for each study was determined by the highest level of bias in any single domain (“worst-domain” approach). Detailed domain-specific ratings are shown in Supplementary Fig. 1.

### Statistical analysis

Statistical software R (version 3.5.1) was used for the analysis^10^. Pooled effect estimates were calculated using random-effects models to account for heterogeneity among studies. Risk Ratios (RRs) with 95% confidence intervals (CIs) were used for binary outcomes. Heterogeneity was assessed using the I² statistic, with thresholds of 25%, 50%, and 75% representing low, moderate, and high heterogeneity, respectively. If heterogeneity persisted (*P* < 0.1, I^2^ > 50%), a sensitivity analysis was performed by systematically excluding each study one at a time to identify the source of heterogeneity. We utilized the inverse variance weighting approach for the pooling of relative risks (RR) and hazard ratios (HR) from each study, based on the reported point estimates and 95% confidence intervals (CIs). The inverse variance method assigns greater weight to studies with more precise estimates, which is determined by the inverse of the variance of the effect size. For each study, the log-transformed effect size (RR or HR) and its associated standard error (SE) were calculated. These log-transformed values and their standard errors were used to compute the pooled log-effect estimate. The inverse variance weight for each study was calculated as the reciprocal of its variance (which is the square of the standard error). The pooled effect size was obtained by calculating the weighted average of the study-specific log-transformed effect sizes, where each study’s weight is proportional to the inverse of its variance. To ensure consistency, all analyses were performed using both log-transformed and raw effect estimates. Equivalent results were obtained when using raw effect estimates and their 95% confidence intervals directly, rather than log-transformed values, confirming the robustness of the meta-analytic findings. Finally, a Baujat plot was used to assess the precision and potential variability in our estimates, providing an intuitive representation of the data distribution^11^. Importantly, a random-effects model including all eligible studies was pre-specified as the primary analysis, in order to account for between-study clinical and methodological heterogeneity. Heterogeneity was explored using Baujat plots and leave-one-out analyses to assess the influence of individual studies on overall estimates. These analyses were performed to evaluate robustness and sources of heterogeneity and were not used as criteria for study exclusion. Additional analyses excluding individual studies were conducted as sensitivity analyses.The level of statistical significance was set to 0.05 (2-tail analysis). Subgroup analyses were conducted to explore potential sources of heterogeneity among Asian versus Western populations. Due to limited number of studies available, it was deemed inappropriate to conduct a funnel plot analysis for evaluating publication bias. Instead, Egger’s regression test was used to assess publication bias, as it provides a statistical evaluation of small-study effects. In accordance with the Cochrane Handbook, we acknowledge that the statistical power of Egger’s test is limited when fewer than ten studies are included; therefore, results should be interpreted with caution, and a non-significant intercept indicates no evidence suggestive of publication bias rather than definitive absence of bias.This test examines asymmetry by regressing the log-transformed risk ratios (log[RR]) against their standard errors, with a non-significant intercept indicating the absence of publication bias^12^.

## Results

### Study selection and characteristics

**Fig. 1:**
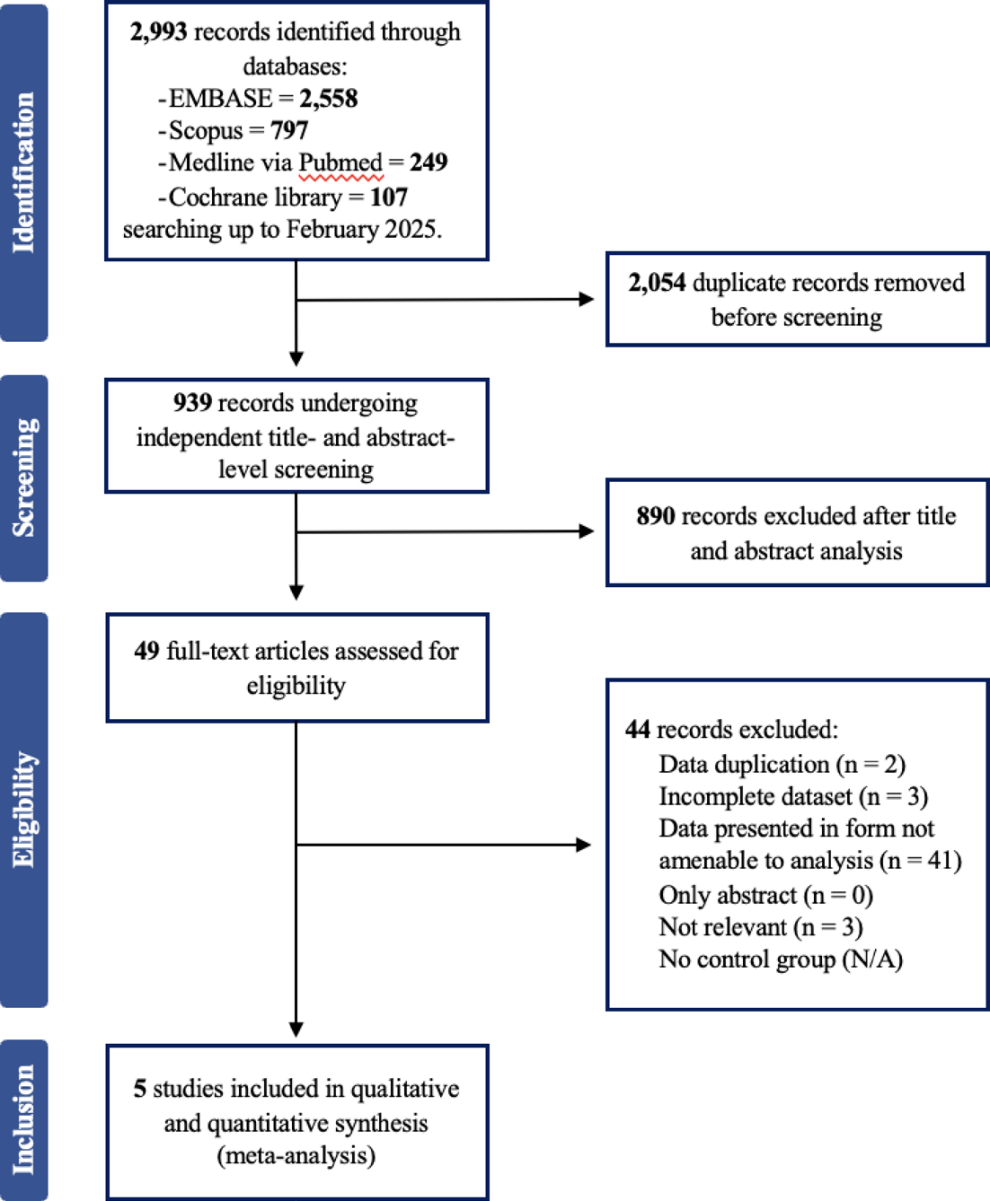
PRISMA flow diagram. A flow diagram representing the selection process of studies included in the meta-analysis, following PRISMA 2020 guidelines. This diagram details the number of studies identified, screened, assessed for eligibility, and included in the final quantitative synthesis.

The study selection process is shown in Fig. 1.

A total of 438 records were identified through database searches. After removal of duplicates and screening, 52 articles underwent full-text review, of which 5 met the final inclusion criteria for this meta-analysis^13–17^ (Table 1).

**Table 1:** Baseline characteristics of included Studies.

First Author	Year	Country	Number of Patients (Mean Age ± SD, % Male)	T2DM (%)	CKD (eGFR < 60 ml/min, %)	Hyperlipidemia (% Statin Therapy, median LDL ± IQR)	Indication for PCI (STEMI, NSTEMI, UA, SA)	Follow-up	Measuring Time (hsCRP)	Key Results
Kalkmann	2018	USA	7026 (64.42 ± 11.06 years, 71.7% male)	51.8%	28.4%	96% (74.5% statin therapy, LDL 82.68 mg/dl ± 34.99)	SA 53.4%, UA 31.6%, NSTEMI 9.8%, STEMI 1.7%	56 weeks (between first and second hsCRP measurement)	Baseline, 1-month post-PCI	Persistent high RIR: 2.6% mortality, 7.5% MI at 1-year, sustained results after adjustment for diabetes, ACS, and LDL
Takahashi	2020	Japan	2032 (66.6 ± 9.7 years, 83.0% male)	44.2%	23.7%	76.5% (74.6% statin therapy, LDL 99.0 [IQR, 81.0–122.0])	SA	4.9 years (IQR, 1.7–9.6) years	Baseline (pre-index PCI) – 6/9 months post-PCI	Persistent high and increased RIR vs. persistent low RIR MACEs HR respectively 2.38 [95% CI: 1.46–3.96] and 2.35 [95% CI: 1.14–4.58]; All-cause death respectively 2.08 [95% CI: 1.41–3.11] and 2.05 [95% CI: 1.13–3.11] after adjustment for age, sex, RIR, HT, CKD, DM, HL, BMI, smoking status, MVD, LVEF, LDL, HDL, triglycerides, use of statins
Ahn	2022	South-Korea	4562 (65.3 ± 11.7 years, 70.6% male)	30.8%	16.2%	53.6% (95.0% statin therapy, LDL 115.9 ± 42.4)	SA 33.6%, UA 9.4%, NSTEMI 31.0%, STEMI 25.2%	36.0 (IQR, 18.9–71.9) years	Baseline – 1 month post-PCI	Persistent high RIR vs. other RIR groups (attenuated and fortified RIR): MACEs HR 1.26 [95% CI: 1.02–1.56]; all-cause death 1.92 [95% CI: 1.44–2.55]; Major Bleeding HR 1.98 [95% CI: 1.30–2.99], adjusted for index MI presentation, age, sex, BMI, smoking, HT, T2DM, HL, CKD, anemia, previous stroke, LVEF, previous PCI for LAD lesion, MVD, potent P2Y_12_ inhibitor, statin, β-blockers and RAAS inhibitors.
Yu	2024	China	1202 (59.5 ± 9.9 years, 75.0% male)	32.8%	6.2%	NA	Hospitalized pts with CHD undergoing planned PCI	12 months	Baseline − 1 month post-PCI	RIR (identified as hsCRP≥1 mg/L) vs. non-RIR (identified as hsCRP<1 mg/L): all-cause death, HR 1.93 [95% CI: 1.21–3.07]. A hsCRP≥2 mg/L cut-off has also been tested, however the HR between groups was not statistically significant.
Gibson	2009	USA	2867 (Age PCI + Atorvastatin group 57.1 ± 10.5, Age CPI + Pravastatin group 57.0 ± 10.9; 79.2% male)	15.8%	NA	21.9% statin therapy, LDL PCI + Atorvastatin group 107 [IQR, 89–128], LDL PCI + Pravastatin group 106 [IQR, 88–127])	UA 24.5%, NSTEMI (37.1%), STEMI (38.4%)	2 years	Baseline – 1 month follow up	High RIR group (PCI Pravastatin 40 mg group) vs. low RIR group (PCI Atorvastatin 80 mg group): composite outcome (death from any cause, MI, documented UA requiring rehospitalization and revascularization at least 30 days after randomization, and stroke)

A summary table presenting key baseline characteristics of the studies included in the meta-analysis. It includes sample size, patient demographics, follow-up duration, and primary outcome measures.

Overall, there were 13,604 patients, 5,833 with high RIR 30 days after PCI and 7,771 in the low RIR group. These studies were conducted across multiple countries, including the USA^14,15^, Japan^16^, South Korea^13^, and China^17^. The mean age of participants ranged from 57.0 ± 10.9 years to 66.6 ± 9.7 years, with a predominantly male population (70.6% to 83.0%). Follow-up durations varied widely across studies, ranging from 12 months^17^ to a median of 5.2 years, providing robust long-term insights into the prognostic implications of RIR. The prevalence of type 2 diabetes mellitus (T2DM) varied across studies, ranging from 15.8%^14^ to 51.8%^15^. Hyperlipidemia was highly prevalent, with statin therapy use ranging from 21.9%^14^ to 95.0%^13^. Median LDL-C cholesterol levels varied from 82.68 ± 34.99 mg/dL^15^ to 115.9 ± 42.4 mg/dL^13^. Indications for PCI included stable angina (SA), unstable angina (UA), non-ST-elevation myocardial infarction (NSTEMI), and ST-elevation myocardial infarction (STEMI). The proportion of STEMI cases ranged from 1.7%^15^ to 38.4%^14^. Regarding RIR definition, we tailored the concept to high RIR vs. low RIR at 30-day follow-up after PCI and excluded the other subgroups including increased RIR (first low-followed by a high hsCRP) and attenuated RIR (first high-followed by a low hsCRP) as described in the manuscript from Kalkmann and cols^15^. All the studies established as hsCRP cutoff ≥ 2 mg/L, however the Japanese group established as high RIR hs-CRP > 0.9 mg/L according to the median value of their cohort^16^.

### Risk of bias assessment

The overall methodological quality of the included observational studies was moderate according to the ROBINS-I assessment as shown in Supplementary Fig. 1. The primary source of bias was residual confounding, as most registries did not account for inflammatory comorbidities or post-PCI treatment differences. Selection bias was also possible because hsCRP measurements were available only for patients with complete laboratory data. In contrast, exposure classification and outcome assessment were generally robust, as hsCRP was measured using standardized assays and outcomes such as mortality and myocardial infarction were registry-verified. Missing data and reporting domains were mostly rated moderate, reflecting incomplete covariate adjustment and absence of pre-registered protocols. None of the studies were judged to be at critical risk of bias. Sensitivity analyses excluding studies with serious risk of bias yielded results consistent with the overall pooled estimates.

### Residual inflammatory risk and MACE

**Fig. 2:**
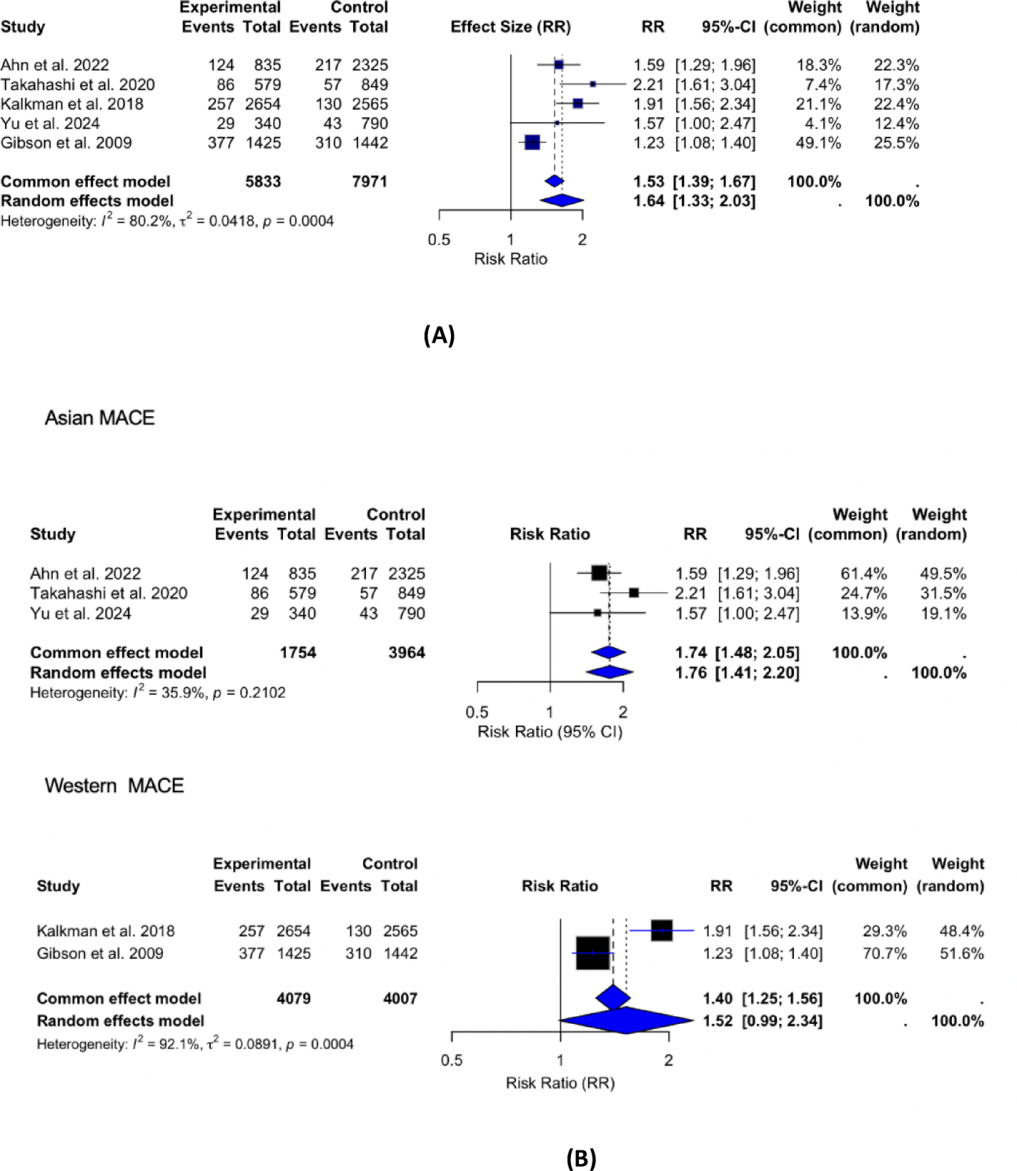
(**A**) Forest Plot for MACE. Forest plot showing the association between residual inflammatory risk (RIR) and major adverse cardiovascular events (MACE) across five studies using a random-effects meta-analysis (I² = 80%). Sensitivity analysis revealed that removal of Gibson et al.^14^ reduced heterogeneity (I² = 11.1%) without changing the overall effect size. (**B**) In subgroup analyses stratified by region (Western vs. Asian cohorts), the association between RIR and MACE was directionally consistent across regions; however, the interaction test was not statistically significant (p for interaction = 0.56), indicating no evidence of effect modification by region.

Data from 5 studies^13–17^ examining the relationship between RIR and MACE revealed substantial heterogeneity among the studies (I^2^ = 80%) (Fig. 2A). In the primary random-effects meta-analysis including all studies, high residual inflammatory risk was associated with an increased risk of major adverse cardiovascular events (RR 1.64, 95% CI 1.33–2.03; I² = 80%).

A sensitivity analysis as well as Baujat plots as shown in Supplementary Fig. 2 were conducted by performing a leave-one-out approach to assess the impact of individual studies on heterogeneity. The removal of Gibson et al.^14^ significantly reduced heterogeneity (I² from 80% to 11.1%), with the Q-test becoming non-significant (*p* = 0.3376). However, the overall effect size remained stable (random-effects RR = 1.91, 95% CI: 1.65–2.22, *p* < 0.0001), indicating that while Gibson et al. contributed substantially to heterogeneity, its inclusion did not alter the overall conclusions confirming the stability of the primary findings.The high heterogeneity among Western studies likely reflects greater variation in metabolic comorbidities, statin use, and timing of hsCRP assessment compared with Asian cohorts, leading to wider dispersion of effect estimates. In addition, to account for the precision of individual studies, we also performed a pooled estimate using inverse variance weighting. This approach gives more weight to studies with larger sample sizes, resulting in a more precise pooled risk estimate. After applying inverse variance weighting, the pooled RR was 1.79 (95% CI 1.61–1.99, *p* < 0.0001), indicating that patients with high residual inflammatory risk had nearly an 80% higher likelihood of adverse cardiovascular events compared with those with low risk.To assess potential publication bias, we performed an Egger’s test using a linear regression of the log of the Risk Ratios (RR) on the standard error of the log(RR) for the five included studies. The regression results revealed that the intercept (β0\beta_0β0​) was 0.3541 (*p* = 0.285) and the slope (β1\beta_1β1​) was 1.1835 (*p* = 0.572). The residual standard error was 0.2406, and the R-squared value was 0.1174, indicating that the model explained only a small proportion of the variance in the log(RR). The lack of a significant slope (*p* = 0.572) suggests that there is no significant relationship between effect sizes and their standard errors, indicating no evidence of publication bias as depicted in Supplementary Fig. 3. When performing subanalysis, high RIR was shown to be less associated with MACE in Western (RR: 1.52, 95% CI [0.99, 2.34], *p* < 0.00001, *I*^2^ = 92%) than in Asian (RR: 1.78, 95% CI [1.52, 2.09], *p* = 0.32, *I*^2^ = 12%). In subgroup analyses stratified by region (Western vs. Asian cohorts), the association between RIR and MACE was directionally consistent across regions; however, the interaction test was not statistically significant (p for interaction = 0.56), indicating no evidence of effect modification by region (Fig. 2B).

### **Residual inflammatory risk and all-cause mortality**

**Fig. 3:**
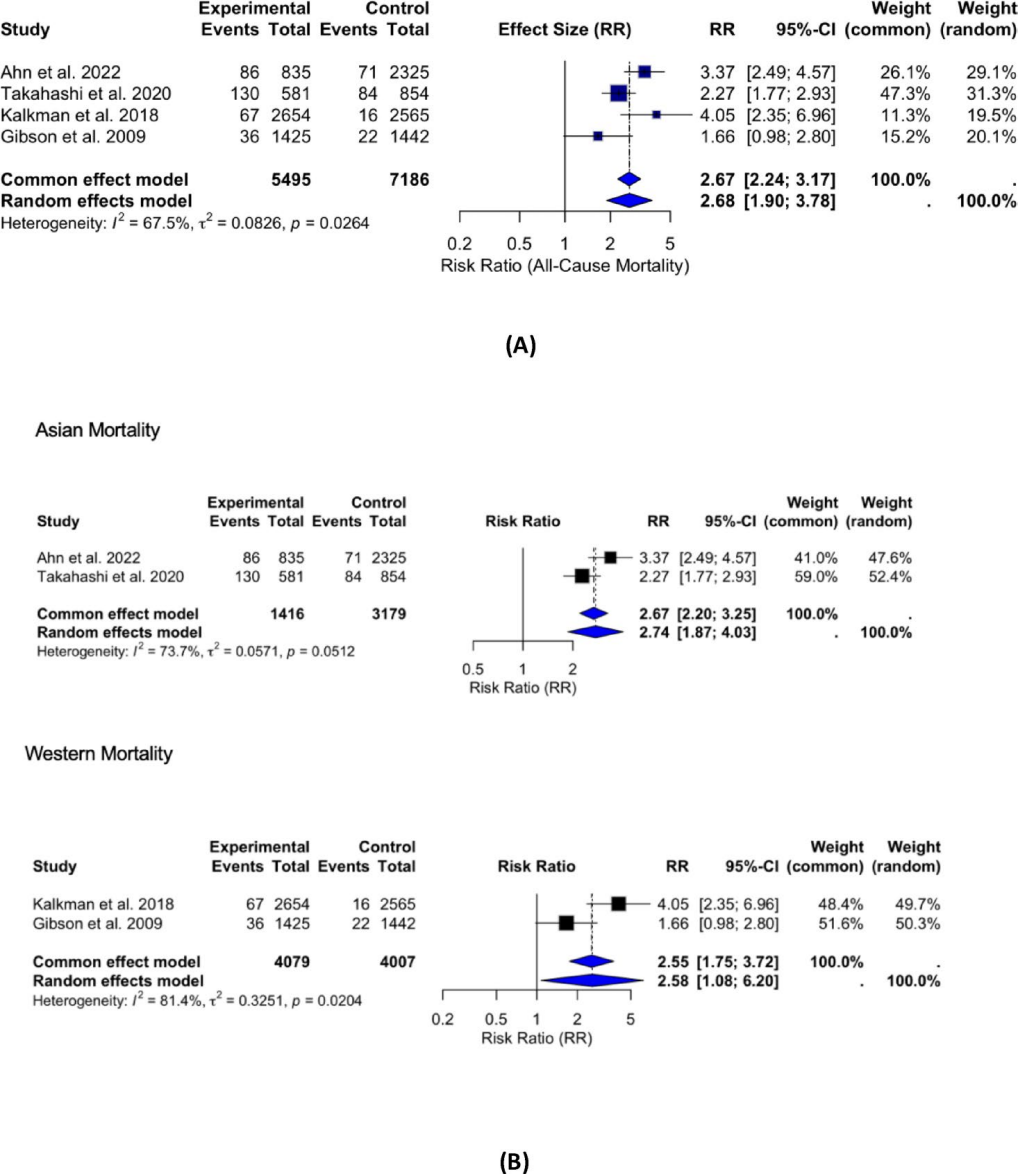
(**A**) Forest plot for all-cause mortality. forest plot showing the association between residual inflammatory risk (RIR) and all-cause mortality across four studies using a random-effects meta-analysis (I² = 67%). Sensitivity analysis using leave-one-out methods indicated that exclusion of Gibson et al. reduced heterogeneity (I^2^ = 34%) without changing the overall effect size. (**B**) Subgroup analysis comparing the association of high residual inflammatory risk (RIR) with all-cause mortality in Western and Asian populations. The association was slightly stronger in Asian populations (RR = 2.74, 95% CI: 1.87–4.03, I² = 73%) than in Western populations (RR = 2.58, 95% CI: 1.08–6.20, I² = 81%), with no statistically significant heterogeneity between groups.

Data from 4 studies^13–16^ examining the relationship between RIR and all-cause mortality revealed substantial heterogeneity among the studies (I^2^ = 67%) (Fig. 3A). In the primary random-effects meta-analysis including all studies, high residual inflammatory risk was associated with an increased risk of all-cause mortality (RR 2.68, 95% CI 1.90–3.78; I² = 67%).

A sensitivity analysis as well as Baujat plots were conducted as shown in Supplementary Fig. 4 by performing a leave-one-out approach to assess the impact of individual studies on heterogeneity. The removal of Gibson et al.^14^ significantly reduced heterogeneity (I^2^ from 67% to 34%), with the Q-test becoming non-significant (*p* = 0.21). However, the overall effect size remained stable (random-effects RR = 3.25 (95% CI: 2.49–4.25, *p* < 0.0001), indicating that while Gibson et al. contributed substantially to heterogeneity, its inclusion did not alter the overall conclusions. The pooled relative risk (RR) for all-cause mortality was 2.93 (95% CI 2.73–3.13, *p* < 0.0001) using inverse variance weighting, indicating that patients with high residual inflammatory risk had nearly a threefold higher risk of death compared with those with low risk. These findings underscore the robustness and clinical relevance of the meta-analytic results. Egger’s regression analysis was conducted to assess publication bias. The intercept of the regression model was not significantly different from zero (*p* = 0.303), and the relationship between the standard error of the log odds ratio and effect size was also not significant (*p* = 0.824), indicating no evidence of publication bias or small-study effects in the included studies as depicted in Supplementary Fig. 5. When performing subanalysis, high RIR was shown to be less associated with all-cause mortality in Western (RR: 2.58, 95% CI [1.08, 6.20], *p* = 0.02, *I*^2^ = 81%) than in Asian (RR: 2.74, 95% CI [1.87, 4.03], *p* = 0.05, *I*^2^ = 73%) (Fig. 3B).

Subgroup analysis comparing the association of high residual inflammatory risk (RIR) with all-cause mortality in Western and Asian populations. The association was slightly stronger in Asian populations (RR = 2.74, 95% CI: 1.87–4.03, I² = 73%) than in Western populations (RR = 2.58, 95% CI: 1.08–6.20, I² = 81%), with no statistically significant heterogeneity between groups.

### Residual inflammatory risk and non-fatal myocardial infarction

**Fig. 4:**
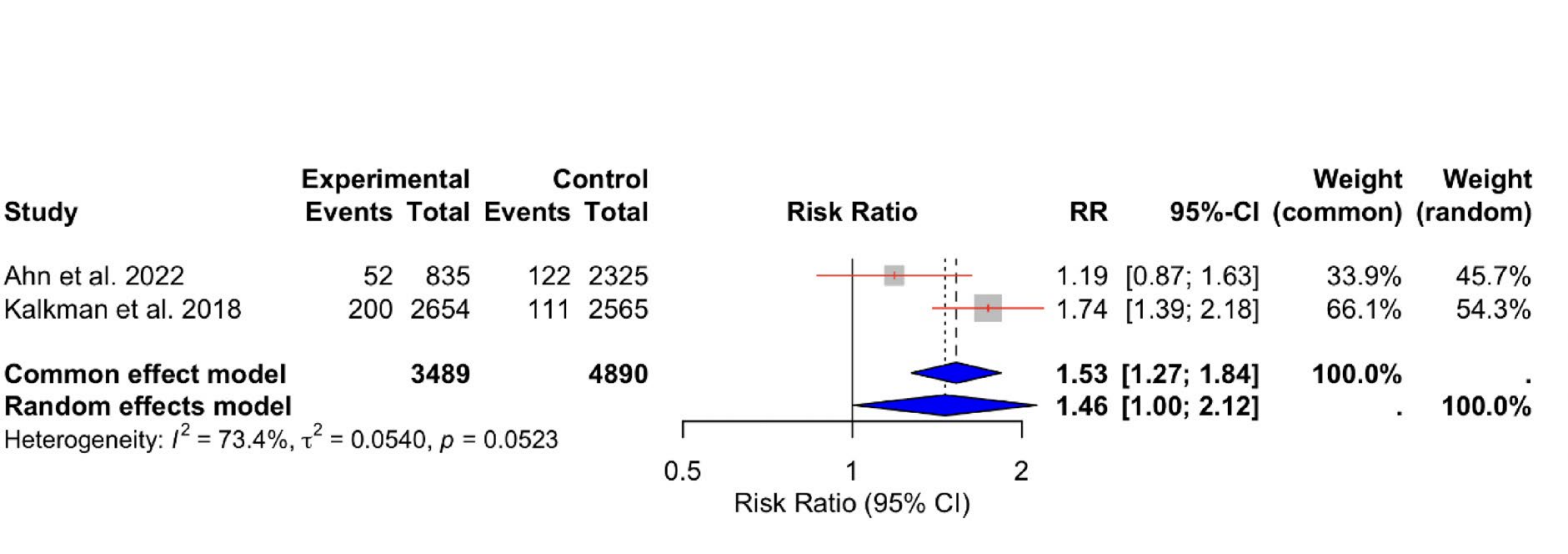
Forest Plot for Non-Fatal Myocardial Infarction. A graphical representation of the pooled risk of non-fatal myocardial infarction among patients with high versus low RIR.

Data from 2 studies^13,15^ examining the relationship between RIR and non-fatal MI revealed substantial heterogeneity among the studies (I^2^ = 73%). The overall effect size was consistent with previously reported outcomes (RR: 1.46, 95% CI [1.00, 2.12] (Fig. 4).

The pooled relative risk (RR) for non-fatal myocardial infarction was 1.56 (95% CI 1.29–1.89, *p* < 0.0001) using inverse variance weighting, indicating that patients with high residual inflammatory risk had approximately a 56% higher likelihood of experiencing a recurrent myocardial infarction compared with those with low risk.

### Residual inflammatory risk and non-fatal stroke

**Fig. 5:**
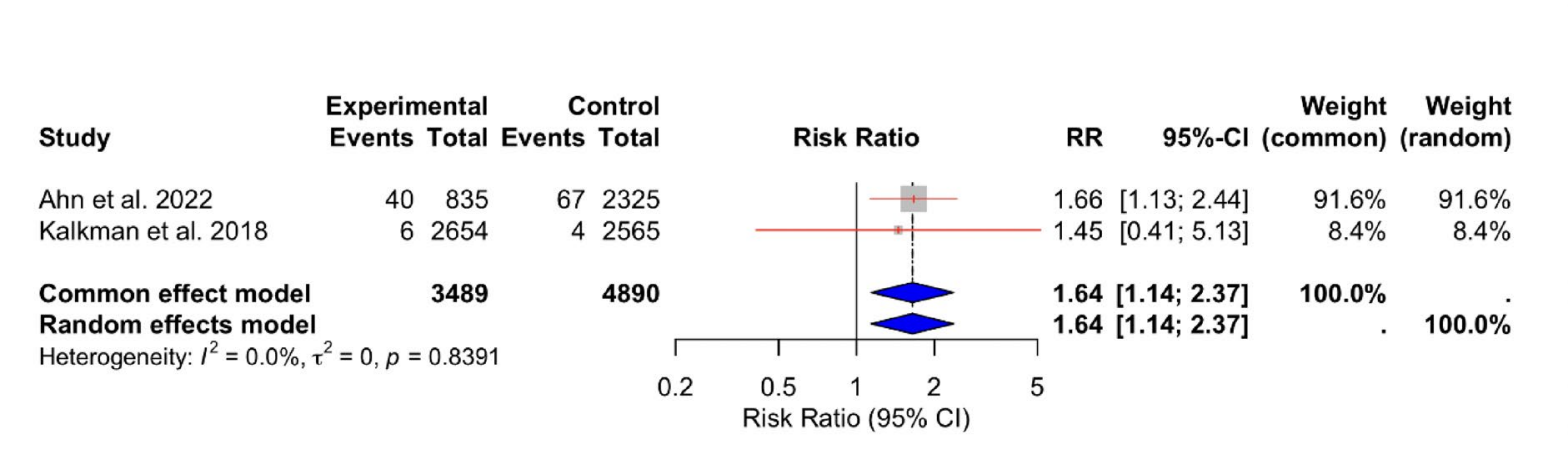
Forest plot for non-fatal stroke. A meta-analysis of the relationship between RIR and non-fatal stroke, showing risk ratios and heterogeneity analysis.

Data from 2 studies^13,15^ examining the relationship between RIR and non-fatal stroke revealed homogeneity among the studies (I^2^ = 0%). The overall effect size was consistent with previously reported outcomes (RR: 1.64, 95% CI [1.14, 2.37] (Fig. 5).

The pooled relative risk (RR) for non-fatal stroke was 1.55 (95% CI 1.17–2.06, *p* < 0.0001) using inverse variance weighting, indicating that patients with high residual inflammatory risk had about a 55% greater risk of experiencing a non-fatal stroke compared with those with low inflammatory risk.

### Residual inflammatory risk and major bleeding

Major bleeding outcomes were reported in two studies^13,15^. In the primary random-effects model, high residual inflammatory risk was not significantly associated with major bleeding (RR 2.10, 95% CI 0.80–5.47; I² = 87%). Given the limited number of studies and substantial heterogeneity, these findings should be considered exploratory and hypothesis-generating only.

## Discussion

In this systematic review and meta-analysis of 5 observational studies including a total of 13,604 patients, we evaluated the prevalence of high RIR in statin-treated patients 30 days after undergoing contemporary PCI and its association with clinical outcomes over a 1-year follow-up period. The key findings can be summarized as follows: (1) over 40% of patients undergoing contemporary PCI were found to have high RIR. These patients were older and exhibited a higher prevalence of risk factors and comorbidities compared to their non-high RIR counterparts; (2) high RIR was independently associated with a higher incidence of MACE, primarily driven by all-cause mortality, followed by spontaneous MI, non-fatal stroke, major bleeding (3) when stratifying the analysis by race, the association between high RIR and MACE remained significant but was more pronounced in studies involving Asian cohorts.

The well-established link between elevated cholesterol levels and the morbidity and mortality associated with ASCVD has positioned LDL-C reduction as a cornerstone of guideline-recommended strategies to improve cardiovascular outcomes^18^. However, as observed in recent randomized control trials, a sizeable proportion of PCI patients remains at increased cardiovascular risk despite achieving target serum LDL-C while on high-intensity statin therapy or combination regimens. This residual risk has spurred efforts to identify additional contributing factors and optimize preventive strategies tailored to individual patient profiles. Among these factors, RIR with hs-CRP widely recognized as its reliable biomarker has emerged as a critical driver of adverse cardiovascular outcomes and a promising target for both primary and secondary prevention strategies^3^. To the best of our knowledge, this is the first comprehensive systematic review and meta-analysis that includes studies evaluating the prognostic impact of persistently high RIR in patients undergoing contemporary PCI.

The first interesting finding we observed is the high prevalence of persistently elevated RIR, exceeding 40% in the pooled cohort, indicating that subclinical inflammation is relatively common in statin-treated patients undergoing PCI. This result is particularly notable, as it aligns with data from major clinical trials that report rates ranging from 30% in patients with well-controlled LDL-C to 40% in those without^19^, and may be even higher in real-world practice due to the lack of routine hsCRP measurement, potentially leading to underestimation in the reported prevalence^4^. Moreover, when studies were stratified by region, differences in the prevalence of high RIR were observed between Western and East Asian cohorts. These differences likely reflect variation in baseline inflammatory profiles, hsCRP thresholds, statin intensity, follow-up duration, and healthcare system factors rather than intrinsic biological or genetic determinants. Importantly, no statistically significant interaction was observed between region and the association of high RIR with major adverse cardiovascular events, indicating no evidence of effect modification by geographic or racial background^20^. Accordingly, subgroup findings should be interpreted cautiously and considered descriptive in nature. Minor variations in hsCRP thresholds used to define high RIR across studies likely reflect population-specific baseline inflammation and methodological differences in study design rather than meaningful biological divergence. Despite these differences, the direction of association between elevated hsCRP and adverse cardiovascular outcomes remained consistent across cohorts, supporting the robustness of the overall findings. Second, we observed that persistently elevated RIR was associated with nearly double the risk of MACE and a 2.5-fold increase in all-cause mortality within 1-year. Although risks for non-fatal MI, non-fatal stroke and major bleeding were also elevated, interpretation is limited by significant heterogeneity, particularly for non-fatal MI, and the low event rates for stroke (~ 1%), which constrain generalizability. In addition, the observed association between RIR and bleeding outcomes is exploratory and insufficient to support causal inference, particularly given the small number of studies, extreme heterogeneity, and lack of statistical significance in the primary random-effects analysis. It is important to highlight that RIR after PCI is a driver of MACE even in patients with baseline low LDL-C (≤ 70 mg/dl)^21^ as well as may act as a risk enhancer in patients with CKD undergoing PCI increasing both MACE and all-cause mortality^22^.

This analysis holds substantial clinical and therapeutic implications. On the diagnostic front, our findigns emphasize the role of hsCRP as a valuable point-of-care marker for RIR. Notably, RIR performed well in other clinical scenarios to predict adverse outcomes. In patients with acute ischaemic stroke or transient ischaemic attack, high RIR increased the risks of 1-year stroke recurrence, composite vascular events, mortality and poor functional outcome as well as vulnerable plaque features in the carotid artery^23,24^.

Moreover, we highlight the importance of assessing baseline pre-PCI inflammatory burden regarless of acute or chronic clinical presentation, but even more critically its post PCI evaluation, which remains strongly associated with poor prognosis. However, inflammation is a complex, multidimensional process that extends far beyond hsCRP measurements. For instance, an interesting Italian observational registry involving 2353 patients undergoing primary PCI evaluated the prevalence of CRP-independent inflammatory patterns and the one-year relationship with adverse clinical outcomes^25^. They determine at baseline and 1-month after PCI the levels of “systemic immune inflammatory index” assessed by both neutrophil-to-lymphocyte ratio (NLR) and platelet-to-lymphocyte ratio. Patients with both persistent-high and up-sloping CRP-independent inflammatory patterns were associated with an increased risk of adverse events at one-year follow-up. These findings are aligned with the Mount Sinai PCI registry including 7.287 patients which demonstrated in patients with baseline NLR > 5 undergoing PCI an increased risk of MACE at 1-year follow-up after adjusting for different clinical variables including hsCRP^26^.

**Fig. 6:**
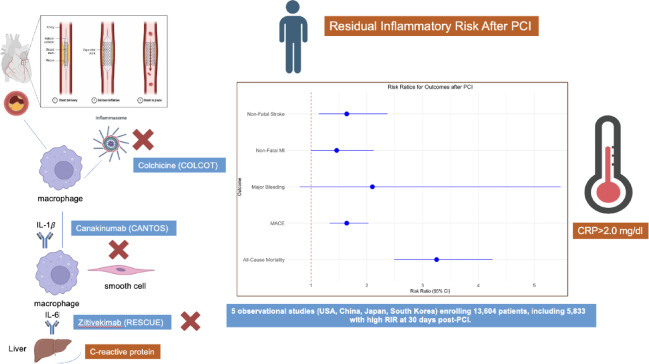
Residual inflammatory risk and potential therapeutic targets. Conceptual framework illustrating the role of residual inflammatory risk (RIR) in post-PCI care, integrating hsCRP assessment and potential downstream biomarkers (interleukin-1, interleukin-6) for comprehensive risk stratification and pharmacological targeting.

Further research is needed to identify complementary biomarkers downstream the inflammatory cascade (interleukin-1, interleukin-6) and clarify their roles in the comprehensive evaluation of RIR (Fig. 6).

On the therapeutic front, our findings fuel the growing evidence supporting the integration of anti-inflammatory therapies with lipid-lowering treatments to improve cardiovascular outcomes.

The NLRP3 inflammasome initiates a cytokine cascade that contributes to the development of atherosclerosis. Its activation triggers the release of interleukin-1β (IL-1β) and IL-6, promoting systemic inflammation. In addition, IL-6 is a downstream cytokine of the NLRP3 inflammasome and a key marker of inflammation. Unlike hs-CRP, Mendelian randomization studies have identified IL-6 as a causal contributor to increased cardiovascular risk^27^. Colchicine mainly but not exclusively blunts inflammatory response by NLRP3 inflammasome pathway inhibition. So far, clinical trials have produced mixed evidence, with COLCOT^28^ and LoDoCo2^29^ trials demonstrating the efficacy of low-dose colchicine (0.5 mg daily) in acute and chronic coronary syndromes whereas the recent CLEAR SYNERGY (OASIS 9) trial, involving over 7,000 patients starting colchicine soon after MI and continued for a median of 3 years, failed to confirm its superiority over placebo, underscoring the critical need for further research into optimizing anti-inflammatory strategies in cardiovascular care^30^. In the PCI patient population, periprocedural myocardial infarction and injury have been associated with increased MACE and mortality^31^. The hypothesis that inflammation could partially contribute to periprocedural MI and injury led to the development of COPE-PCI^32^ and COLCHICINE-PCI^33^ randomized clinical trials. The COPE-PCI randomized 196 patients to 1 mg followed by 0.5 mg colchicine 6 h to 24 h pre-PCI versus placebo. There was significantly lower periprocedural myocardial injury in the colchicine group and no differences in periprocedural MI. In addition, the COLCHICINE-PCI study randomized 400 patients undergoing PCI to 1.8 mg of colchicine 6 h to 24 h pre-PCI or placebo. There were no differences in the primary endpoint of periprocedural myocardial injury or MI. Both IL-6 and hs-CRP levels were significantly reduced in the colchicine group. The COL BE PCI trial^34^ (NCT06095765) enrolling 2,770 patients in 19 sites in Belgium will provide further data regarding the use of low dose colchicine in patients with both acute and chronic coronary syndromes undergoing PCI. All patients will have baseline hsCRP measurements and a Second Manifestations of Arterial Disease (SMART) risk score calculation. The primary endpoint is the time from randomization to the first occurrence of a composite endpoint consisting of all-cause death, spontaneous non-fatal myocardial infarction, non-fatal stroke, or coronary revascularization. Finally, ongoing research is exploring innovative therapeutic targets within the inflammatory cascade. One such target is interleukin-6 (IL-6), with ziltivekimab^35^, a human monoclonal antibody against IL-6 ligand, currently under investigation. The ARTEMIS trial (NCT06118281) is a double blind, sponsor-driven, randomized controlled enrolling globally around 10.000 patients with AMI to either ziltivekimab or placebo. The first loading dose is administered within the first 36 h for STEMI and 72 h for NSTEMI and then once monthly by subcutaneous injection. The study will provide further insights whether IL-6 downregulation may improve cardiovascular outcomes after AMI.

This study has limitations. First, the number of eligible studies was relatively small, reflecting the limited availability of investigations that prospectively assessed RIR using serial hsCRP or equivalent biomarkers after PCI. Nevertheless, the total pooled sample size exceeded 13,600 patients, providing adequate power for the primary analyses. Importantly, effect estimates were consistent across subgroups and sensitivity analyses, and heterogeneity was acceptable, supporting the robustness of our findings. Similar meta-analyses in emerging cardiovascular biomarker research have also included a modest number of high-quality studies while yielding clinically meaningful conclusions. Secondly, the definition of high RIR varied across studies, with hsCRP thresholds reflecting population-specific distributions rather than a universal biological cutoff. As such, the pooled estimates should be interpreted as reflecting relative inflammatory risk within each cohort, limiting extrapolation to absolute hsCRP values across populations. Moreover, concomitant anti-inflammatory therapies were not systematically reported across studies, representing a potential source of unmeasured confounding that may influence both hsCRP levels and clinical outcomes.

**Fig. 7:**
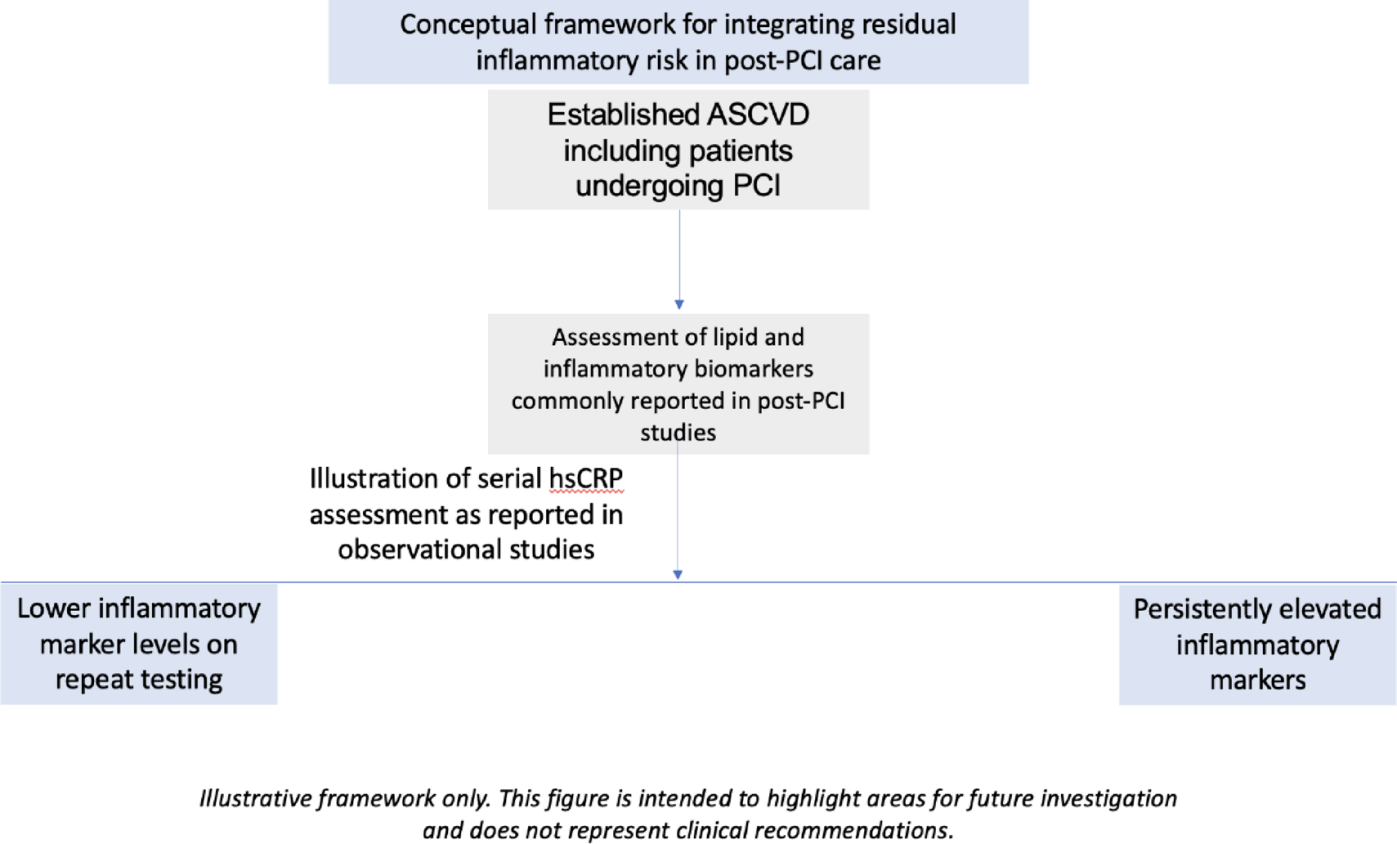
Conceptual framework for future RIR-targeted research after PCI. Conceptual framework for the evaluation of RIR following PCI, depicting serial hsCRP measurements and potential stratification based on persistent inflammation. The figure is intended to highlight areas for future investigation rather than to guide clinical decision-making.

Future research is still warranted to understand the contributions of various domains of residual ASCVD risk, beyond RIR and LDL-C control, to enable the development of tailored risk stratification approaches and more comprehensive preventive strategies. Translating these findings into practice, serial hs-CRP assessment after PCI could be integrated into secondary prevention follow-up, complementing lipid parameters and lifestyle modifications to identify patients with persistent RIR despite optimal LDL-C control^36^. We outline a hypothesis-generating framework that highlights potential pathways through which RIR could be evaluated in future studies of statin optimization, anti-inflammatory therapies (such as colchicine or IL-6 pathway inhibition), and follow-up assessment strategies (Fig. 7).

Our recommendations are in line with the inflammation and cardiovascular disease scientific statement just published by the American College of Cardiology (ACC)^37^. Beyond IL-6 and NLRP3, other biomarkers such as GlycA^38^ and lipoprotein-associated phospholipase A₂ (Lp-PLA₂)^39^ merit investigation for refining inflammation targeted risk assessment and therapy.

## Conclusions

Our systematic review and meta-analysis of five observational studies highlights the critical role of RIR following PCI in predicting long-term outcomes. Despite successful revascularization, persistent inflammation remains a significant contributor to the development of MACE and all-cause mortality. These findings suggest that RIR serves as an important prognostic factor and underscores the need for targeted strategies aimed at reducing inflammation in the post-PCI setting. Future prospective studies should investigate the efficacy of anti-inflammatory treatments in mitigating RIR and improving patient outcomes.

## Supplementary Information

Below is the link to the electronic supplementary material.

Supplementary Material 1

Supplementary Material 2

Supplementary Material 3

Supplementary Material 4

Supplementary Material 5

Supplementary Material 6

Supplementary Material 7

## Data Availability

The data supporting the findings of this study are available within the manuscript and its supplementary information files. If additional data are required, they can be made available upon reasonable request to the corresponding author.

## Abbreviations

PCI: Percutaneous coronary intervention
MACE: Major adverse cardiovascular events
RIR: Residual inflammatory risk
STEMI: ST-elevation myocardial infarction
AMI: Acute myocardial infarction
LDL-C: Low-density lipoprotein
RCT: Randomized controlled trials
CI: Confidence interval
Hs-CRP: High sensitivity C-reactive protein
ASCVD: Atherosclerotic cardiovascular disease
