# Supplementary material for: Residual inflammatory risk and clinical outcomes after contemporary percutaneous coronary intervention: a systematic review and meta-analysis
Source: Sci Rep. 2026 Feb 12;16:8584. doi: 10.1038/s41598-026-39691-1 (PMC12976032; doi:10.1038/s41598-026-39691-1)
Supplement: Supplementary file 1 — Supplementary Material 1 [file 41598_2026_39691_MOESM1_ESM.docx]

### **Supplementary Material**

**Supplementary Table 1. PRISMA 2020 Checklist.**
Detailed reporting checklist outlining adherence to PRISMA 2020 guidelines for systematic reviews and meta-analyses.

**Supplementary Figure 1. ROBINS-I risk of bias assessment.**
Graphical summary of risk of bias across included studies, evaluated using the ROBINS-I tool. Domains include confounding, selection of participants, classification of interventions, deviations from intended interventions, missing data, outcome measurement, and selection of reported results.

**Supplementary Figure 2. Baujat plot (MACE).**
Baujat plot illustrating influence of individual studies on heterogeneity for the outcome of major adverse cardiac events (MACE).

**Supplementary Figure 3. Egger’s regression test for publication bias (MACE).**
Egger’s regression plot assessing small-study effects for studies reporting major adverse cardiac events (MACE).

**Supplementary Figure 4. Baujat plot (all-cause mortality).**
Baujat plot showing study influence and heterogeneity contribution for analyses of all-cause mortality.

**Supplementary Figure 5. Egger’s regression test for publication bias (all-cause mortality).**
Egger’s regression plot assessing small-study effects for all-cause mortality outcomes.
