# Supplementary figures and images for: Residual inflammatory risk and clinical outcomes after contemporary percutaneous coronary intervention: a systematic review and meta-analysis
Source: Sci Rep. 2026 Feb 12;16:8584. doi: 10.1038/s41598-026-39691-1 (PMC12976032; doi:10.1038/s41598-026-39691-1)

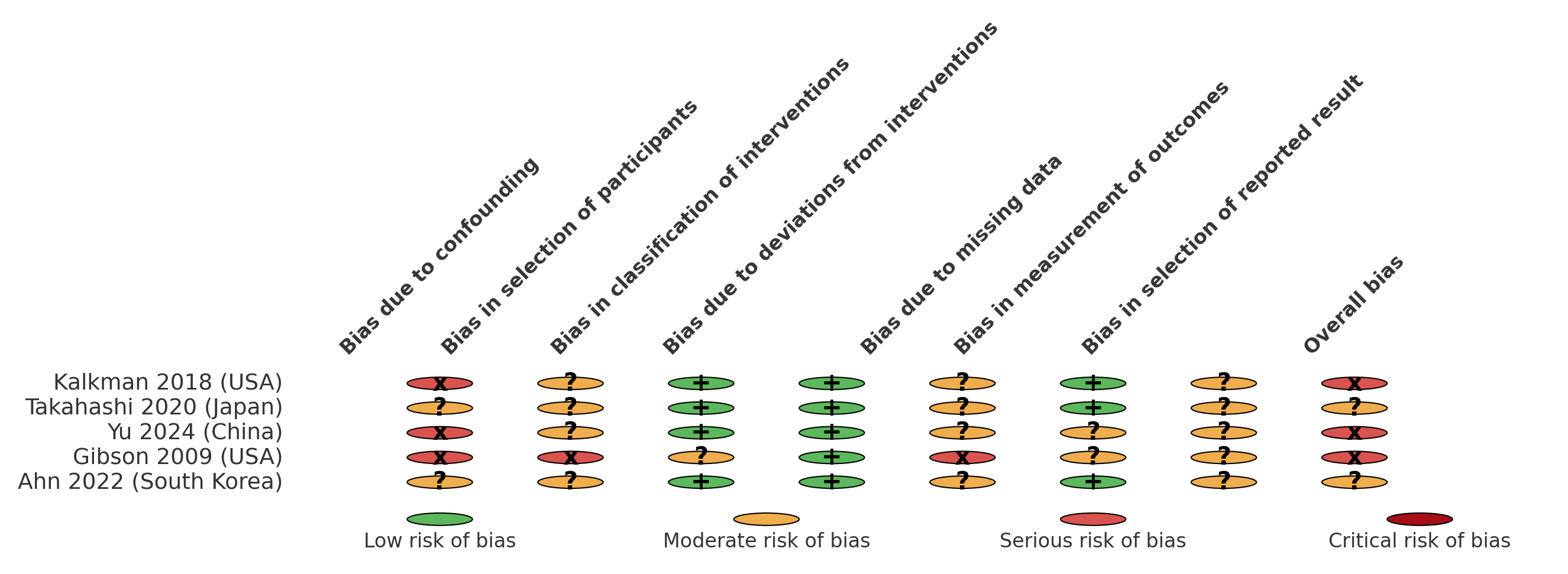

Supplement: Supplementary file 2 — Supplementary Material 2 [file 41598_2026_39691_MOESM2_ESM.tiff]

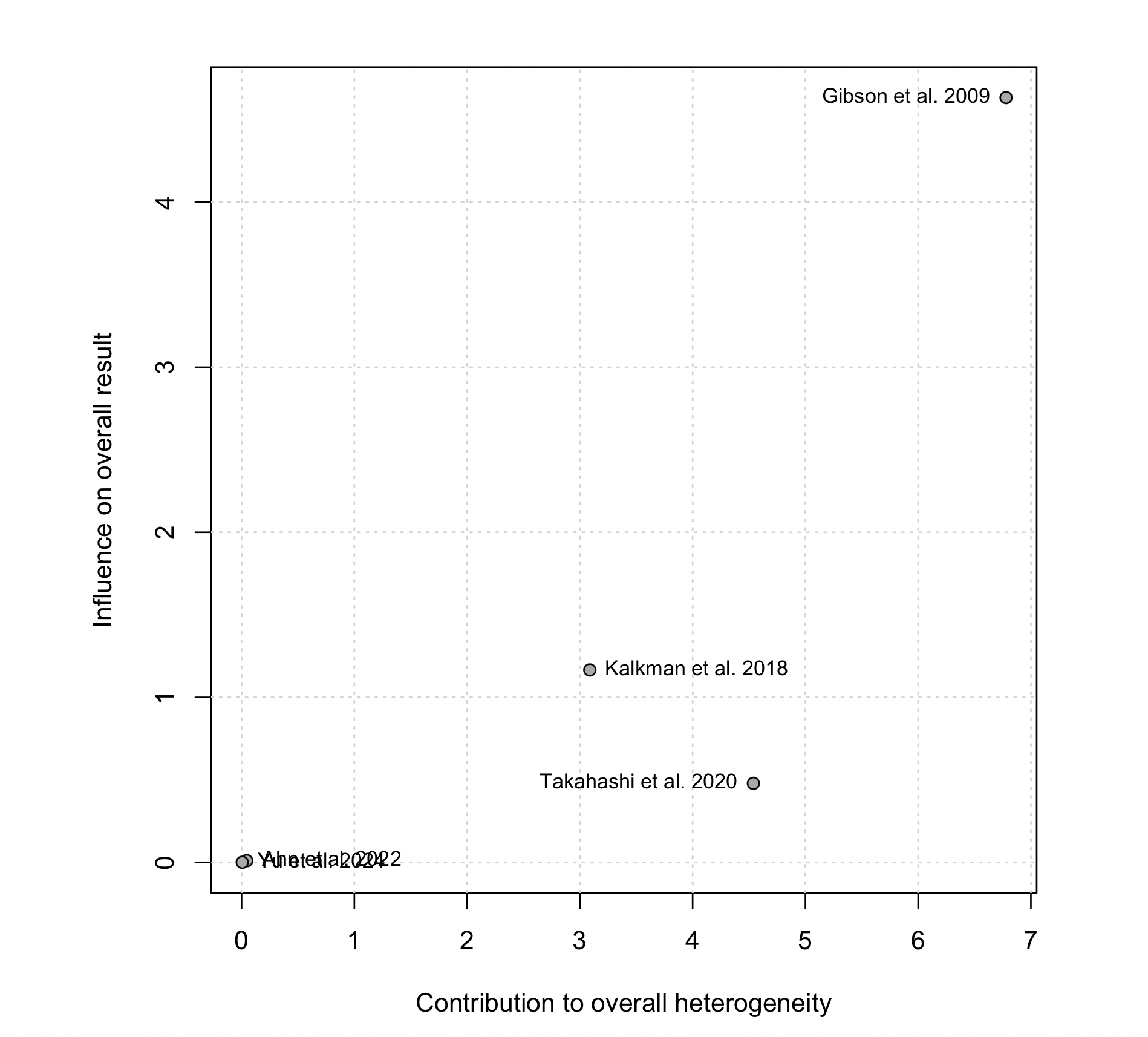

Supplement: Supplementary file 4 — Supplementary Material 4 [file 41598_2026_39691_MOESM4_ESM.tiff]

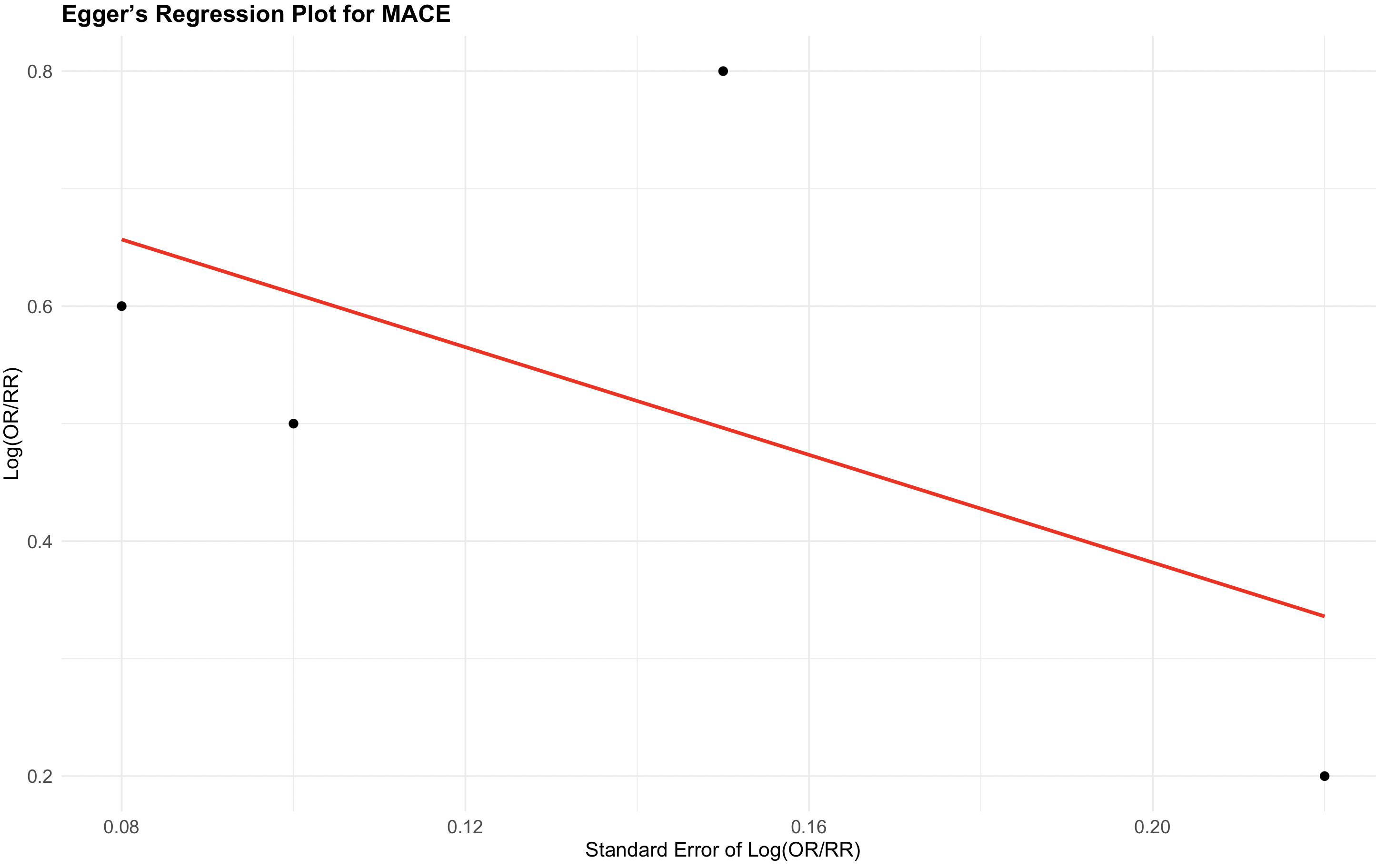

Supplement: Supplementary file 5 — Supplementary Material 5 [file 41598_2026_39691_MOESM5_ESM.tiff]

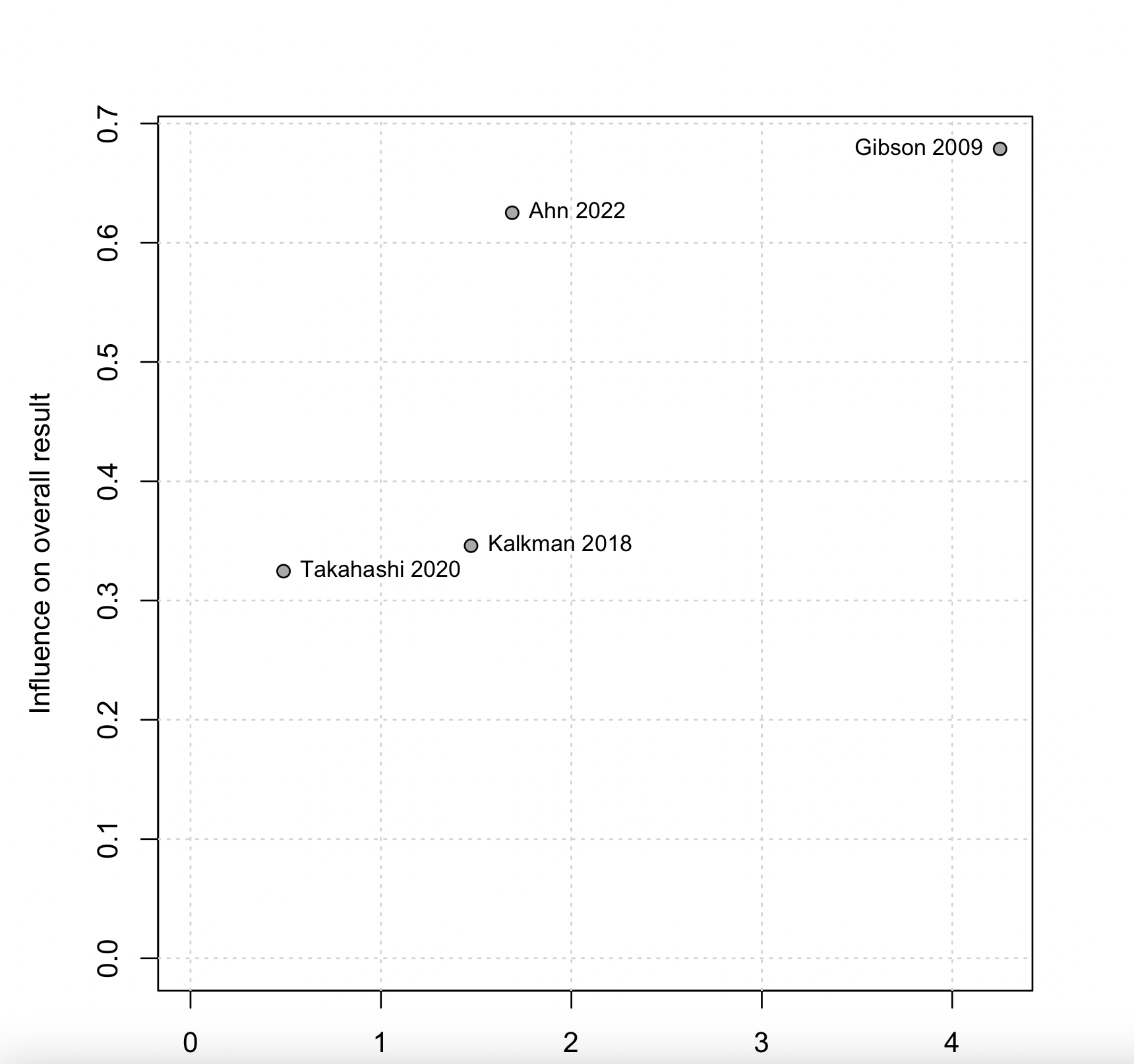

Supplement: Supplementary file 6 — Supplementary Material 6 [file 41598_2026_39691_MOESM6_ESM.tiff]

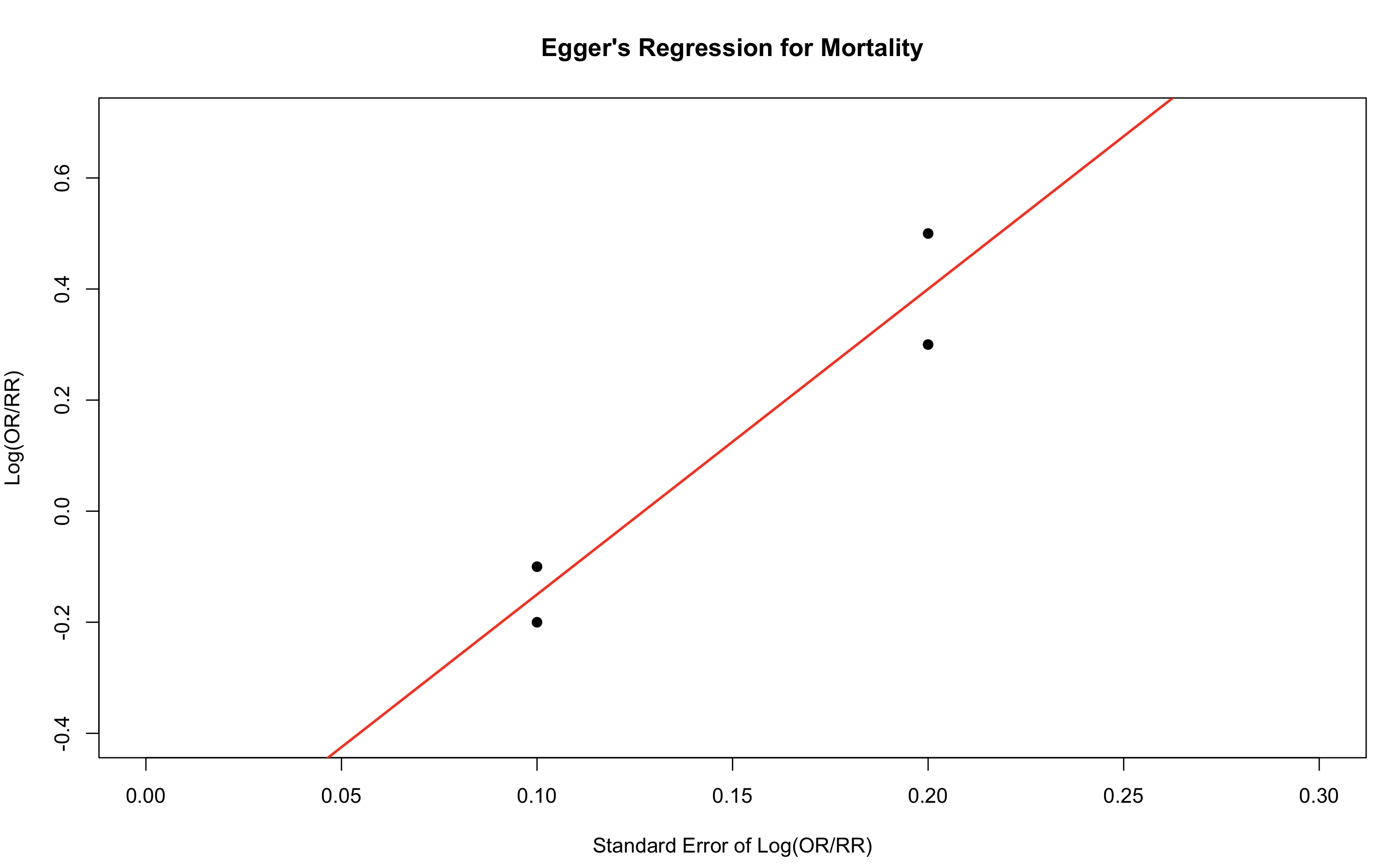

Supplement: Supplementary file 7 — Supplementary Material 7 [file 41598_2026_39691_MOESM7_ESM.tiff]
